# Selective Impact of Early Parental Responsivity on Adolescent Stress Reactivity

**DOI:** 10.1371/journal.pone.0058250

**Published:** 2013-03-13

**Authors:** Daniel A. Hackman, Laura M. Betancourt, Nancy L. Brodsky, Lara Kobrin, Hallam Hurt, Martha J. Farah

**Affiliations:** 1 University of Pennsylvania, Department of Psychology, Center for Neuroscience and Society, Center for Cognitive Neuroscience, Philadelphia, Pennsylvania, United States of America; 2 The Children's Hospital of Philadelphia, Division of Neonatology, Department of Pediatrics, Philadelphia, Pennsylvania, United States of America; Johns Hopkins University School of Medicine, United States of America

## Abstract

Research in animals has shown that early life experience, particularly parenting behaviors, influences later-life stress reactivity. Despite the tremendous relevance of this finding to human development and brain function, it has not been tested prospectively in humans. In this study two aspects of parenting were measured at age 4 in a sample of healthy, low socioeconomic status, African American children, and stress reactivity was measured in the same children 11–14 years later using a modified version of the Trier Social Stress Test (*n* = 55). Salivary cortisol was measured before, during and after the stressor and data were analyzed using piecewise hierarchical linear modeling. Parental responsivity, independent of the use of physical discipline, was positively related to cortisol reactivity. Effects were independent of subjective appraisals of the stressor and were also independent of other environmental risk factors and current psychosocial functioning. Therefore this study demonstrates in a novel and precise fashion that early childhood parental responsivity prospectively and independently predicts stress reactivity in adolescence.

## Introduction

Parental effects on cognitive and socioemotional development are hypothesized to be due, in part, to the influence of early childhood parental care on stress reactivity [1]–[4]. In addition, by shaping the nature of the response to stress and challenge, such an influence is also likely to determine the impact of other environmental factors across development [5] and thus have a broad and enduring influence across time. However, despite the importance of the hypothesis that early parenting influences later-life stress reactivity in humans, support is indirect.

The limbic hypothalamic-pituitary-adrenal (HPA) axis is one such stress response system which mobilizes in response to physical or psychological threats to well-being and facilitates homeostatic regulation through change [4], [6], [7]. Stressful events activating the HPA axis result in a molecular cascade that eventually increases circulating glucocorticoids, cortisol in humans, which in turn provide a signal for deactivation of the system through receptors in the hippocampus. In contrast with the sympathetic adrenomedullary system, which rapidly mobilizes resources and facilitates the fight or flight response, the HPA axis is mobilized more slowly in response to acute stressors and induces longer term changes, in part through changes in gene expression that broadly influence the brain and other organ systems.

Animal research has demonstrated lasting effects of parenting on later-life stress reactivity. Indeed, these effects are sometimes referred to as “programming,” a term that emphasizes the critical, lasting effects of early parenting behaviors [1], [8]–[10]. In rodents, the offspring of mothers who exhibit high levels of licking, grooming and arched-back nursing (which facilitates pups' access to milk) show increased hippocampal glucocorticoid receptor expression, enhanced negative feedback regulation, decreased hypothalamic corticotropin-releasing factor (CRF) expression, more modest HPA-axis responses to stress, and less fearful behavior. Maternal care is a causal factor in this case, as demonstrated by cross-fostering studies [1], [8]–[10]. Studies with nonhuman primates in which maternal responsiveness is experimentally decreased find higher levels of CRF in the offspring [11]. Although identifying a human analogue of these behaviors is challenging, they are often considered analogues of affective components of caregiving, such as sensitivity and responsivity [4]. However, differences between species as well as the difficulty in extrapolating parenting characteristics across species highlights the need to investigate this question in humans.

Evidence in humans shows that a range of atypical rearing experiences are related to HPA-axis reactivity to stress. These experiences include abuse or maltreatment [12]–[14], maternal depression [15] and experiences of parenting in the context of parental death [16]. This literature, however, is complex and yields contradictory findings [17], suggesting the effects of exposures to adversity and caregiving on stress reactivity are non-linear in nature [5], [18]. If true, then a focus on atypical caregiving, such as abuse and psychopathology, cannot provide a full account of the effects of parenting on reactivity. We must also study the effects of parenting behaviors across a broad range of typical parenting behaviors. Moreover, it is such variation in typical caregiving behaviors related to warmth and responsivity that has been the focus of the animal literature just cited [4].

In order to test the hypothesis that parental responsivity and sensitivity have an enduring relationship with HPA-axis reactivity to stress, a prospective longitudinal study is necessary. This is because cross sectional studies, in which parenting behavior and stress reactivity are assessed at the same stage of development [19]–[26], and very short-term prospective designs [27]–[30] do not distinguish between effects of parenting on the concurrent and near-term state of the child's stress physiology and the programming phenomenon revealed by animal research. The same limitations apply to the effect of parenting interventions assessed in the near-term with preschoolers [31]–[33]. Although retrospective assessments of parenting concurrent with a later-life measurement of stress reactivity avoid that problem [34]–[36], they instead encounter the problem of retrospective bias, whereby individuals with less well regulated stress systems may be more inclined to remember their parents in a negative light.

The present study was designed to test the hypothesis that variation in a broader range of typical parenting behaviors in early childhood has a lasting relationship with HPA axis reactivity to stress. In particular, this study aimed to test the hypothesis that those behaviors related to warmth and responsivity, independently from other parenting behaviors such as behavioral control and physical discipline [37], have a specific and enduring association with stress reactivity. We therefore took advantage of an on-going longitudinal study of a cohort of low-socioeconomic status (SES), African Americans who have been studied from birth through adolescence [38]. The Parental Responsivity and Acceptance of Child subscales from the Early Childhood Home Observation Measurement of the Environment (HOME scale), administered when the children were 4 years old, were used as indicators of caregiver warmth and responsiveness and discipline [39] and chosen as indices of the affective and control components of caregiving [37]. Between the ages of 15 and 18, participants underwent exposure to a mild social stressor, a modified version of the Trier Social Stress Test (TSST) [40], [41], while salivary cortisol was measured across the protocol. To assess the specificity of the effect of parental responsivity, independent of correlated factors, analyses include other measures of early experience, life stress, and psychosocial development that were collected between birth and adolescence. Consequently, this study is uniquely positioned to determine, prospectively, if parental responsivity has an enduring and independent association with stress reactivity.

## Materials and Methods

### Participants

Participants were 55 African-American adolescents from the control group of a larger longitudinal study of prenatal cocaine exposure who participated in the stress reactivity protocol [38], [42]. Participants were recruited at birth from a single inner-city hospital, born at or near term (≥ 34 weeks) with no serious medical conditions. Mothers were native English speakers and had no past or present indication of major psychiatric illness as determined by medical chart review and interview at time of enrollment. None of the children were exposed prenatally to cocaine as confirmed by maternal self-report, medical chart review, and maternal and infant urine drug screens. Women were excluded if they used substances other than cigarettes, marijuana or alcohol. None of the infants had Fetal Alcohol Syndrome. All families were receiving public assistance and mothers had a high school education or less at the time of birth. Since enrollment, participants have completed semi-annual evaluations.

The characteristics of the sample are depicted in Table 1. Four participants who completed the stressor protocol were excluded due to missing data on aspects of early parenting. One participant did not sleep the night before and was thus excluded from the analysis, while another was excluded because cortisol values were greater than 3 *SD* above the mean of the other measurements on 6 of 9 samples. Analyses thus included a total of 49 participants (28 female, 57.1%) between the ages of 15 and 18 (*M* = 16.7, *SD* = 1.1). Written consent was obtained from participants aged 18 and older or from the parents or guardians of participating children, who also gave assent to participate. The Institutional Review Boards of the University of Pennsylvania and The Children's Hospital of Philadelphia approved the project.

**Table 1:** Sample characteristics.

	Mean±SD
Sex (female)	28 (57)^a^
Age at HOME evaluation (years)	4.1±0.1
Age at stress protocol (years)	16.7±1.1
Gestational Age (weeks)	38.9±2.1
Birth weight (kg)	3.1±0.6
Parental education at birth (years)	11.5±1.1
HOME total score (Age 4)	43.9±5.5
HOME subscales (Age 4)	
arental Responsivity	5.2±1.2
cceptance	3.6±0.8
Primary caregiver (Age 4)	
other	45 (91.8)^a^
ther family	3 (6.1)^a^
nrelated	1 (2.0)^a^

a Number (percent)

The original cohort consisted of 119 control participants enrolled between 1989 and 1992, and the majority of attrition occurred by the time participants were 30 months of age [38], largely related to inadequate number of study personnel secondary to fiscal constraints [43], [44]. In the current study, 46% of the original control participants were retained, a number that has been stable for the past 8 years [43], . Control children lost to follow-up did not differ from those who participated in the stress protocol on gender (*p* = .14) level of prenatal care (*p* = .70), maternal age at birth (*p* = .89), gestational age (*p* = .77), birth weight (*p* = .17), head circumference (*p* = .68), prenatal exposure to alcohol (*p* = .84), cigarettes (*p* = .80) or marijuana (*p* = .75), or performance on the Bayley Scales of Infant Development at 6 (*p* = .54), 12 (*p* = .39), 18 (*p* = .12), or 24 (*p* = .89) months of age. It is unlikely that any protocol-specific attrition is due to aversion to novelty or to stress, as participants were familiar with the study environment and personnel. In addition, compared with the 62 control participants who attended any other session in the five years prior to the current session, 88.7% were retained in the current study.

### Measures of early parenting

Parenting behavior was assessed using subscales from the Home Observation for Measurement of the Environment (HOME) [39], administered at age 4 (*M* = 4.1, *SD* = 0.1). The HOME is a 1-hour semi-structured interview and observation that generates eight subscales. The current analysis focused on two subscales from the HOME that best measure the affective and control aspects of parenting in contrast to the type of environmental enrichment offered in the household: the Parental Responsivity subscale (e.g. ‘parent holds child close 10–15 minutes per day,’ ‘parent converses with child at least twice during visit’, ‘parent caresses, kisses, or cuddles child during visit’, ‘parent answers child’s questions or requests verbally’) as well as the Acceptance of Child subscale, which primarily measures the use of physical discipline (e.g. ‘parent does not scold or derogate child more than once,’ ‘parent neither slaps nor spanks child during visit’, ‘no more than one instance of physical punishment during past week’). Table 1 reports the total and subscale scores for the HOME. Parental Responsivity and Acceptance at age 4 were not correlated (*r* = .22, *p* = .13), indicating that these subscales measure distinct aspects of early experience. At the time of assessment most participants (91.8%) were in the care of their mother (Table 1). To examine differential attrition, HOME subscale scores for those in the stress protocol were compared with the 18 control participants who completed the HOME scale but were lost to follow-up by the time of the stress protocol. There were no differences in the Parental Responsivity subscale, *t*(67) =  −1.33, *p* = .19; however, there was a differences in scores on the Acceptance subscale, *t*(65.8) = 2.49, *p* = .02. Notably, Acceptance subscale scores had greater variability and were lower overall in those who participated in the stress protocol. Consequently, there was greater use of physical discipline and variability in this behavior in those who remained in the study than in those who did not complete the protocol.

### Stressor Protocol and Procedure

**Figure 1:**
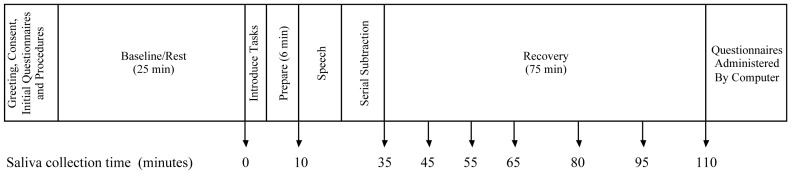
Timeline of procedures. Reproduced from [74] with permission.

Participants completed a modified version of the Trier Social Stress Test (TSST) [40], [41], [45], [46] designed to induce a moderate level of stress. The protocol for the TSST is outlined in Figure 1. In order to increase social evaluation [45] participants underwent the protocol in groups of 2 (*n* = 18, 36.7%) or 3 (*n* = 31, 63.3%) [41], [46], matched for sex. All sessions began between 11:30am and 1:30pm to control for the diurnal pattern of cortisol, with 91.8% of sessions at 1:00pm or 1:30pm. Participants were contacted the evening before their session and instructed to avoid consuming a major meal 60 minutes before their session, drinking milk or eating other dairy products 30 minutes before the session, eating acidic or high sugar foods 20 minutes before the session, brushing teeth within 1 hour before their session, and consuming alcohol in the 12 hours prior to the session (Salimetrics, LLC, State College, PA).

Upon arrival, participants were greeted by the experimenter and directed to sit in a semi-private room where they were able to interact with an experimenter but not with other participants. After completion of the consent process, experimenters conducted a short interview to assess participant compliance with pre-appointment instructions and to survey use of prescribed and non prescribed medications. To establish a baseline prior to stress induction and prior to the first collection of a saliva sample, participants watched a video with minimally arousing content for 25 minutes. Participants then performed stressor tasks in a testing room with other group members and an unfamiliar experimenter dressed in a lab coat acting as a judge and directing the testing room activities. To further enhance the social evaluative component of the stressor [45], [47] participants were told their performance was being videotaped and scored. They were all simultaneously given 6 minutes to prepare a 3-minute speech promoting their candidacy for a summer job, and they each gave their speech facing the video camera, the other participants, and the judge. After all participants completed their speech, they were subsequently instructed to perform serial subtraction by eights, aloud, for 3 minutes, in front of the same audience. Individuals were given unique four-digit numbers as starting points. If subtraction mistakes were made they were told to re-start from the beginning. Order of participation for each task, relative to the other participants in the group, was determined randomly. The duration of the stressor was thus 6 minutes shorter for those in groups of 2 as opposed to 3. After all participants completed the stressor task they returned to the semi-private room for a 75 minute recovery period where they watched the remainder of the videotape started during baseline. After the recovery period, questionnaires were completed via audio computer assisted self-interview (ACASI) using MediaLab 2004.3.24 (Empirisoft Software, New York, NY).

### Measures of the Stress Response

Salivary cortisol was the primary outcome of interest, and as outlined in Figure 1 saliva samples were collected at nine different times: at baseline after completion of the 25 minute video, after speech preparation, at the completion of stressor tasks for all members of the group (approximately 35 minutes after baseline), and 10, 20, 30, 45, 60 and 75 minutes after stressor completion. Collection times were identical for all participants. Saliva samples were collected using the passive drool technique and were frozen immediately at −70°C. They were transported on dry ice to Salimetrics, LLC (State College, PA) for analysis using enzyme immunoassay techniques. The test uses 25 µl of saliva per determination, has a lower limit of sensitivity of 0.003 µg/dl and standard curve range from 0.012 to 3.0 µg/dl. Intra- and inter-assay coefficients of variation were 3.5% and 5.1% respectively. Assays were conducted in duplicate and average cortisol concentrations were used. To correct for skewed distributions the natural log of the average cortisol concentration was our outcome measure.

Subjective responses during the stressor as well as retrospective appraisals were also assessed. Participants rated their anxiety level using a seven-point Likert-type scale (1 =  very calm and relaxed; 3 = feeling pretty calm and relaxed; 5 = a little bit nervous, but not too bad; 7 = very nervous or stressed) concurrent with the collection of saliva samples. After completion of the stressor, participants were asked how stressful and challenging they found the speech and math tasks, with response choices structured along a seven-point Likert type scale (1 = Not at all challenging or stressful, 7 = Extremely challenging or stressful).

### Control Variables

Measures of neonatal health, methodological factors, and additional participant behaviors that may affect stress responses were included in analyses to control for potential confounds. Participants' birth weight and gestational age were obtained by chart review at the time of enrollment [38] (see Table 1). Prenatal substance exposure was coded for analysis on an integer scale of 0–3, with 1 point for each of the following substances: tobacco, alcohol, and marijuana. Prenatally, 11 participants were exposed to cigarettes (22.4%), 5 to alcohol (10.2%), and 2 to marijuana (4.1%); 8 (16.3%) were exposed to one substance prenatally, 2 (4.1%) were exposed to 2 substances, and 2 (4.1%) were exposed to 3 substances. Participants' use of prescription and over-the-counter medications was also assessed [48]. Of all classes of medications, only use of oral contraceptives was reported by more than two participants (*n* = 3) and thus included as a potential control variable in analyses [49]. Five participants currently smoked cigarettes (10.2%). Mean hours of sleep the night before was 7.8 (*SD* = 2.4) while the average time participants had been awake at the beginning of the protocol was 5.4 hours (*SD* = 2.0).

### Additional Measures of Environmental Risk and Psychosocial Functioning

Other assessments were employed to determine if the effects of parental responsivity and acceptance were independent of other aspects of childhood risk factors as well as psychosocial functioning (see Table 2). Measures included stressful events over the lifetime, as reported by the participant at the time of the stressor protocol using the Life Events Checklist [50]. Child abuse or neglect was ascertained via experimenter interviews with the primary caregiver and participant across all waves of the longitudinal study and was coded as “yes” for any report of abuse and neglect by an adult caregiver over the course of the study. Violence exposure was measured using the Things I Have Seen and Heard questionnaire [51], a 20-item questionnaire concerning violence exposure at home and in the community, administered to children at 10.7 years of age (*SD* = 1.0) [42].

**Table 2:** Additional environmental risk factors and psychosocial functioning.

	Mean±SD
Environmental Risk Factors	
Violence Exposure (*n* = 46)	10.3±7.3^a^
Abuse / Neglect	6 (12.2) b
Life Stress	10.2±6.7^c^
Psychosocial Functioning	
Depression, BDI-II	8.2±7.5^d^
Internalizing Behavior, YSR (*n* = 48)	47.8±9.4^e^
Externalizing Behavior, YSR (*n* = 48)	49.0±9.8^e^
Coping, Task-focused	56.6±9.1^e^
Coping, Emotion-focused	47.3±11.6^e^
Coping, Avoidance-focused	59.1±8.7^e^
Mastery	12.7±3.2^f^

a The Things I Have Seen and Heard [51] scale ranges from 0 to 80

b Number (percent)

c Life stress scale [50]: Number of items endorsed as present, ranging from 0 to 32

d From the BDI-II [52]: 0–13, minimal; 14–19, mild ; 20–28, moderate ; 29–63, severe

e Standardized T-scores, *M* = 50, *SD* = 10

f Ranges from 7 to 28, with lower scores representing higher mastery

On the day of the session psychosocial questionnaires were administered to participants after completion of the TSST. Current symptoms of psychopathology were measured using the Beck Depression Inventory, 2^nd^ edition (BDI-II) [52], and the internalizing and externalizing scales of the Youth Self Report (YSR) of the Achenbach System of Empirically Based Assessment (ASEBA) [53]. In addition, in order to rule out the effects of non-clinical psychological characteristics, self-reports were obtained for perceived mastery over the environment, using the Pearlin Mastery Scale [54], and tendencies towards task-, avoidance-, and emotion-focused coping, as measured with the Coping Inventory for Stressful Situations [55].

### Data Analysis

Our primary analytic strategy was piecewise hierarchical linear modeling, a strategy that allows distinct modeling of different phases of change over time, thus permitting the separate modeling of reactivity and recovery phases following administration of a stressor [20], [56]. With a piecewise approach, a Level-1 model is estimated that represents the individual change in salivary cortisol across the protocol and includes both fixed components and random components (intercept and slopes) that are permitted to vary across individuals. Time was recoded into two separate Level-1 components to create a two-piece linear model. The first component represents time linearly from baseline through the measures of cortisol taken ten minutes after the completion of the stressor (minute 45), capturing the episode of reactivity to the stressor given both the duration of the stressor and the delay between stress response and the secretion of cortisol into saliva. Saliva collection times outlined in Figure 1 for the reactivity episode were thus coded, in minutes, as 0, 10, 35, 45, 45, 45, 45, 45, and 45. The second linear component represents the episode of recovery from the stressor, the time from 10 minutes after the completion of the stressor through the end of the protocol. Saliva collection times for the recovery episode were thus coded as 0, 0, 0, 0, 10, 20, 35, 50, and 65. This results in the following Level-1 model: 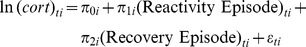



In this model, the outcome is the natural log of salivary cortisol, and π_0i_ represents the intercept, π_1i_ represents the slope during the reactivity episode and π_2i_ represents the slope during the recovery episode. Due to the coding scheme employed the intercept, π_0i_ is an estimate of baseline salivary cortisol before administration of the stressor.

Several steps were taken to ensure that the piecewise modeling approach fit the data well and was not biased. Different group sizes and differences in task participation order, influencing both stressor duration and onset/offset, might result in systematic differences in the timing of peak cortisol levels. However, employing Fisher's Exact Test (due to small cell sizes), there was no relationship between the time of peak cortisol and either group size (*p* = .68) or order of participation in tasks (*p* = .85). Second, the linear piecewise model was compared to a quadratic model of change, using both the Akaike Information Criterion (AIC) and the Bayesian Information Criterion (BIC). In the quadratic model participant-specific collection times were used, such that each individual's unique collection profile and stressor duration was accounted for, without assumptions about the timing of reactivity and recovery. Should a piecewise model be mis-specified due to systematic differences in collection times and timing for reactivity and recovery, this alternative quadratic model should be a better fit for the data. However, this was not the case – the piecewise model was superior to the quadratic model. First, a quadratic model was only able to converge with a random intercept, linear component and fixed quadratic effect, while a piecewise model included a random component for recovery. Consequently, only the piecewise model allowed prediction of individual differences in recovery in addition to reactivity and baseline. Second, the piecewise model was a better fit to the data as measured by both AIC (piecewise = 229.1, quadratic = 351.1) and BIC (piecewise = 242.4, quadratic = 358.6). In summary, the piecewise model fits the data well and does not appear to suffer from any systematic mis-specification.

Employing the piecewise model, Level-2 models were also estimated in which the variance in the intercept and slope parameters at Level-1 are predicted by person-level predictors that do not vary across the time of the stressor protocol. The Parental Responsivity and Acceptance subscales were the primary independent variables examined for all Level-2 equations:
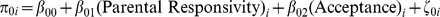


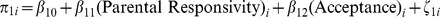


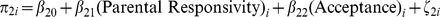



Analyses were conducted in HLM6 [57] using full maximum likelihood estimation. All Level-2 variables included in analyses were grand-mean centered. Forty-six participants (93.9%) had complete data for salivary cortisol at Level 1, as 3 participants each were missing one data point, for a total of 438 observations at Level 1.

The first step in analyses was to establish if there was significant variability across individuals in the random effects for the intercept, slope during reactivity, and slope during recovery that warranted prediction by person-level predictors, such as the Parental Responsivity and Acceptance subscales. To do so an unconditional linear piecewise growth model was created, that accounts for within-individual clustering of cortisol across time without additional second-level predictors, to determine if there is variability across individuals in the parameter estimates for the intercept and slopes during reactivity and recovery. Subsequently, the independent variables, the Parental Responsivity and Acceptance subscales, were added to the model. To identify potential control variables we employed a step-wise procedure, adding variables individually to this model and noting each variable which was significant at a trend level of p<.10 and for which the fit of the prediction model was improved [58]. These variables were sex, age, group size, task performance order, current cigarette smoking, hours of sleep the night before, hours since awakening, prenatal substance exposure, birth weight, gestational age, and use of oral contraceptives. Next, we created a prediction model including all variables identified in the previous step and then removed non-significant (p>.05) control variables sequentially starting with the highest p-value, until only significant control variables remained. Subsequent analyses utilized the resulting model to determine if findings were independent of the environmental risk factors and aspects of psychosocial functioning noted above and in Table 2. Descriptive data were analyzed using SPSS 20.0 (IBM: New York, NY).

## Results

### Subjective Appraisal of the Stressor

Peak anxiety ratings during the stressor were in the moderate range (*M* = 4.5, *SD* = 1.5), while the change in rating from baseline to peak averaged 2.4 (*SD* = 1.6) on the 7-point Likert scale. After stressor administration, both the math (*M* = 4.7, *SD* = 1.8) and speech (*M* = 4.7, *SD* = 1.6) tasks were rated as moderately challenging on the 7-point scale, while the overall protocol was rated as moderately stressful (*M* = 4.2, *SD* = 2.0).

### Variability in Cortisol Reactivity

The unconditional piecewise growth model for cortisol level over time across the protocol yielded a non-significant, positive fixed effect for the reactivity episode (*B* = 0.0026, *p* = .17) and a significant fixed effect for the recovery episode, in the negative direction (*B* =  −0.0085, *p*<.001). However, the random effects for the intercept (σ_0_^2^ = 0.21, *SE* = 0.047, *p*<.001) estimating baseline cortisol before the stressor, slope of the reactivity episode (σ_1_^2^ = 0.0015, *SE* = 0.00003, *p*<.001), and slope of the recovery episode (σ_2_^2^ = 0.0003, *SE* = 0.00001, *p*<.001) were all significant. Consequently, there is significant variation between subjects to support the modeling of systematic individual differences in intercept, reactivity, and recovery.

### Parental Responsivity: Specific predictor of reactivity

**Figure 2:**
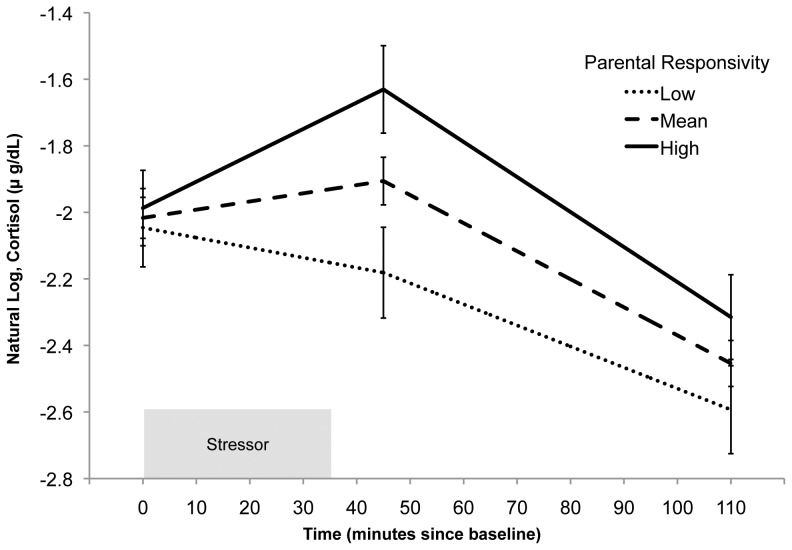
Predicted cortisol reactivity by level of parental responsivity in early childhood. Model-based graphs of cortisol concentration across the stressor protocol by level of parental responsivity. This model controls for the effects of the Acceptance subscale, age, and sex. For illustrative purposes this figure illustrates the predicted cortisol concentration at the mean level of parental responsivity and at two representative values indicative of high and low parental responsivity within the sample, 1.5 *SD* above and below the mean. Error bars represent standard error.

Table 3, Model A illustrates the effect of the Parental Responsivity and Acceptance subscales on cortisol reactivity controlling for sex and age. Increased scores on the Parental Responsivity subscales predicted steeper positive slope during the reactivity episode (*B* = 0.004, *p* = .02, *r_effect_* = .33), while the Acceptance subscale did not predict reactivity (*B* = −0.002, *p* = .24, *r_effect_* = .17). Effects were independent of group size during the stressor, task performance order, participant cigarette smoking, hours of sleep the night before, prenatal substance exposure, birth weight, gestational age, and use of oral contraceptives, which did not meet criteria for inclusion in the model as predictors of intercept, the slope of the reactivity episode, and the slope of the recovery episode. These effects are also independent of the hours since awakening when the first sample was taken, which was a significant predictor of the intercept when included alone (*B* = −0.08, *p* = .02, *r_effect_* = .36) but was not significant (*B* = −0.05, *p* = .09, *r_effect_* = .26) when age and sex were included in the model. Neither the Parental Responsivity nor Acceptance subscales were predictors of the intercept (all *p*>.32) or slope during recovery (all *p*>.11). Figure 2 is based on Model A and depicts the relationship between the Parental Responsivity subscale and cortisol reactivity and recovery. Following Aiken and West [59], we selected values of Parental Responsivity that are 1.5 *SD*'s above and below the grand mean to illustrate this effect in Figure 2: Lower scores on the Parental Responsivity subscale, in contrast with higher scores, predict little or no cortisol response across the protocol.

**Table 3:** Linear piecewise model of salivary cortisol: fixed effects estimates and pseudo-R^2^.

Parameter	Model A			Model B		
	*B*	*p*	*r*_effect_	*B*	*p*	*r*_effect_
Initial Status, π_0i_						
Intercept	−2.02	<.001	.98	−2.02	<.001	.99
Parental Responsivity	0.02	.76	.05	0.09	.16	.23
Acceptance	0.06	.33	.15	0.06	.35	.15
Sex (Female = 1)	0.34	.01	.37	0.46	.002	.50
Age	0.15	.01	.37	0.17	.003	.47
Life Stress				−0.01	.15	.24
Coping: Task-focused				−0.01	.12	.25
Coping: Avoidance				−0.02	.04	.33
Episode 1, Reactivity, π_1i_						
Intercept	0.002	.10	.24	0.002	.16	.22
Parental Responsivity	0.004	.02	.33	0.003	.04	.32
Acceptance	−0.002	.24	.17	−0.002	.15	.23
Sex (Female = 1)	−0.01	<.001	.54	−0.01	.001	.52
Coping: Task-focused				0.0003	.06	.26
Violence Exposure				−0.0004	.10	.23
Episode 2, Recovery, π_2i_						
Intercept	−0.008	<.001	.83	−0.009	<.001	.85
Parental Responsivity	−0.001	.11	.23	−0.002	.10	.26
Acceptance	0.001	.12	.23	0.001	.15	.23
Age	−0.002	.02	.34	−0.002	.02	.36
Life Stress				0.00003	.81	.04
Violence Exposure				0.0002	.07	.29
Externalizing				0.0002	.09	.27
*R_ε_* ^2^	0.67			0.66		
*R_0_* ^2^	0.23			0.47		
*R_1_* ^2^	0.47			0.53		
*R_2_* ^2^	0.33			0.33		

Note: Model B (*n* = 45) includes all potential alternative risk factors or psychosocial predictors that were significant or trend-level significant when added to Level-2 equations in Model A.

### Independence from subjective response

The specific effects of Parental Responsivity on cortisol reactivity may reflect differences in subjective appraisals of the stressor, and thus multiple indices of the subjective response to the stressor were added to Model A in Table 1. Self-rated anxiety, measured concurrently with salivary cortisol, was added as a Level-1, time-varying covariate. However, time-varying anxiety was not a significant predictor of salivary cortisol (*B* = −0.009, *p* = .37, *r_effect_* = .04), while the Parental Responsivity subscale remained a predictor of reactivity (*B* = 0.004, *p* = .02, *r_effect_* = .33) in this model.

In addition, the increase in anxiety from baseline to peak during the stressor, the appraisal of the stressor upon completion, and difficulty ratings of the speech and math components of the stressor, were added as Level-2 predictors of reactivity and recovery. None were predictive of slope during the reactivity (all *p*>.2) or the recovery (all *p*>.31) periods. The Parental Responsivity subscale remained a significant predictor of reactivity in all of the above models (all *p*≤.03).

### Independence from alternative risk factors and current psychosocial functioning

The specific effects of early Parental Responsivity on cortisol reactivity may reflect exposures to other risk factors during childhood or to current psychosocial functioning. Consequently, a series of models were run adding alternative risk factors and indices of symptoms of psychopathology as well as non-clinical traits related to coping strategies and the subjective sense of control over the environment.

In all such models the Parental Responsivity subscale remained a significant predictor of slope during the reactivity period (all *p*≤.04), indicating that the effects were specific. Life stress (*B* =  −0.02, *p* = .04, *r_effect_* = .30), task-focused coping (*B* =  −0.02, *p* = .002, *r_effect_* = .46), and avoidance-focused coping (*B* = −0.03, *p* = .001, *r_effect_* = .51) were significant predictors of intercept. Abuse and neglect, violence exposure, emotion-focused coping, mastery, depressive symptoms, internalizing symptoms, and externalizing symptoms were not predictors of intercept (all *p*>.10). Violence exposure (*B* = −0.0005, *p* = .04, *r_effect_* = .31) and task-focused coping (*B* = 0.0003, *p* = .06, *r_effect_* = .28) were significant and trend level predictors of slope during reactivity, respectively. All other risk factors and psychosocial functioning variables did not predict reactivity (all *p*>.24). Violence exposure (*B* = 0.0003, *p* = .04, *r_effect_* = .31) was a significant predictor of recovery, while externalizing symptoms (*B* = 0.0002, *p* = .07, *r_effect_* = .27) and life stress (*B* = 0.0002, *p* = .07, *r_effect_* = .27) predicted recovery at the trend level. All other risk factors and psychosocial functioning variables did not predict recovery (all *p*>.21).

To confirm that the effects of the Parental Responsivity subscale were independent of all measured risk and psychosocial factors, a subsequent model was run including all significant and trend-level predictors identified in the individual models described above (see Table 3, Model B). When simultaneously including all such predictors, increased Parental Responsivity remained a specific and selective predictor of increased slope of cortisol change during the reactivity period (*B* = 0.003, *p* = .04, *r_effect_* = .32).

## Discussion

This prospective study demonstrates that parental responsivity in early childhood has an enduring association with reactivity to stress. Lower levels of parental responsivity during early childhood were associated with blunted cortisol reactivity to a laboratory stressor in adolescence. To our knowledge this is the first longitudinal study to test the specific association between typical early caregiving experiences, and parental responsivity in particular, and later-life stress reactivity in adolescence or beyond.

The association between parental responsivity and cortisol reactivity is highly specific, as indicated by five aspects of the results. First, behaviors that communicate responsivity and warmth, such as responding verbally to a child's speech and holding the child close 10–15 minutes per day, predict cortisol reactivity while aspects of parental control and a physical approach to discipline, as indicated by items such as scolding, slapping or spanking the child during the visit, do not. Second, the effect of parental responsivity is independent of other significant predictors of the stress response or HPA function in general, such as sex [49], age, cigarette smoking [60], sleep [61], group size and order of task performance during the stressor protocol. Third, the effect of parental responsivity is not explained by differences in the subjective appraisal of the stressor. Fourth, the effect is independent of other markers for early adverse experience, such as birth weight and gestational age [62], prenatal substance exposure [12], childhood abuse or neglect as reported by the child or primary caregiver [12]–[14], [17], as well as other possible environmental risks, including violence exposure and current life stress. Fifth, the effect of parental responsivity on cortisol reactivity is not a function of current psychosocial functioning, including depressive symptoms [63], internalizing or externalizing behavior [64], mastery, or coping style [65]. Parental responsivity in early childhood, over and above these potential alternative pathways, is thus a specific predictor of the cortisol response to stress.

Blunted cortisol reactivity likely increases risk for poor psychosocial outcomes in the future. Blunted or reduced reactivity is a predictor of externalizing behavior and poorer executive function [64], [66]. Parenting interventions that reduce aggressive behavior are mediated, at least in part, via increases in cortisol reactivity to stress [32]. In addition, acutely elevated cortisol is important for mobilizing resources to effectively cope with stress [6], and thus a blunted response suggests such resources are not being mobilized.

The finding that low levels of parental responsivity predict a blunted cortisol response is consistent with the attenuation hypothesis, which posits that chronic stress results in the eventual down-regulation of stress response systems, placing individuals at risk for socioemotional difficulties [3]. However, in the absence of repeated measures over time in the current study we cannot know whether down-regulation has taken place. The direction of the association between parental responsivity and cortisol reactivity is also consistent with the theory of Biological Sensitivity to Context and the Adapative Calibration Model of stress responsivity [5], [18]. According to these accounts, very high levels of support and enrichment as well as very high levels of conflict and adversity promote biological reactivity to context, while moderate support and stress promote lower levels of reactivity. In this study high levels of parental responsivity may be interpreted as buffering the effect of a moderately stressful, low-SES environment. High support in the context of a moderately stressful environment promotes reactivity to the environment as compared with moderate support in the context of moderate stress. It is this difference in severity of risk and support that may explain the discrepancy between the direction of the effects found in this study, which are consistent with those found in some observational and intervention studies [26], [30], [32], [35] but not others [20], [22]. Nevertheless, interpretations in the context of such non-linear models require considerable caution, as there is no consensus on what types of support or adversity should be considered as “exposures”, if these exposures should be combined additively or interactively, or what constitutes high, moderate, or low levels of exposure.

Consistent with the animal literature, we found an association between early life parental responsivity and later stress reactivity. However, the direction of this relationship is opposite to that typically reported in the animal literature. In rodent models longer separations and decreased maternal nurturing behaviors typically predict increased reactivity, while brief separations and manipulations that increase sensitive, nurturing caregiving promote decreased, but not blunted reactivity [1], [8]–[10], though this is not always the case [67]. There are a number of reasons for such inconsistency.

First, equating parental behaviors across species is challenging, not only because of differences in the behaviors themselves, but also because of differences the timing, length, and severity of exposure possible with experimental animal studies and longitudinal observational human studies [10]. Differences between dimensions of human parenting [37] such as sensitivity and responsivity as compared to control and discipline are difficult to translate into animal behavior. Second, human studies have the limitation that important characteristics of the environment and social context are correlated with parental behavior. Some of these characteristics, such as enrichment and unpredictability, are known to influence stress reactivity in animals [68]. Third, gene-environment interactions [69] may render comparisons across genetically controlled animal models and typical human samples difficult. Fourth, differences may also arise due to the differences in stressor paradigms used, and the degree to which they evoke stress responses. In humans, the most effective stressor paradigms incorporate an aspect of social evaluation in addition to uncontrollability [45] that does not have a ready analogue in animal stressor paradigms. Fifth, it may be that the nature of such effects genuinely differ across species, as effects are not all consistent across different animal models [10]. Consequently, extrapolation of inferences from animal models to humans must be done very cautiously and with an awareness of both the methodological and intrinsic differences between the study of animal and human parenting.

A limitation of the present study is that all the adolescents were from low-SES African-American families, raising questions concerning generalizability. African-Americans often demonstrate less robust responses to laboratory-based stressors [70], suggesting that studies in other racial and ethnic groups may have even greater power to detect variance in reactivity. SES correlates with many childhood experiences that may influence HPA axis function, such as cumulative stress [71]. However, the effect of parental responsivity on stress reactivity in this study was independent of indices of early health and development, such as birth weight, as well as self-reported life stress and violence exposure. Consequently, it is unlikely that the effect of parental responsivity on cortisol reactivity is limited only to this population. Nevertheless, future studies are needed to determine if this effect remains in middle- or high-SES families, as both aspects of social support and environmental adversity are likely to influence reactivity [5].

As with all observational studies, it is impossible to firmly establish the direction of causality. It remains possible that genetic differences in stress reactivity influence parenting behaviors, with more reactive individuals also being more responsive parents and transmitting higher cortisol reactivity to their children genetically. However, multiple lines of evidence suggest that parental responsivity is a causal factor. First, although elevated reactivity in children predicts aspects of maternal control and discipline prospectively, it does not predict warmth [72], and the present study found that aspects of parenting related to responsivity and warmth but not control and discipline predicted cortisol reactivity. Moreover, early reactivity in childhood does not predict later attachment [22]. Second, animal literature experimentally demonstrates the effect of environment on stress reactivity [8], though as noted above, extrapolations across species must be undertaken with caution. Third, a parenting intervention has, in randomized controlled trial, demonstrated that change in parenting can impact cortisol reactivity in offspring in the short-term [32]. Lastly, twin studies suggest that environmental factors are the significant primary determinants of the stress response to the first exposure to a stressor [73]. Consequently, it is likely that parental responsivity is influencing offspring stress reactivity.

In summary, early childhood parental responsivity selectively predicts cortisol reactivity to a social stressor in adolescence, over and above the effects of discipline, early developmental markers, life stress, violence exposure, and current psychosocial functioning. To our knowledge, this study provides the first prospective evidence in humans that early parental responsivity can predict stress reactivity more than a decade later. The present findings converge with experimental evidence from animal models and suggest that parental responsivity has enduring effects on neurobiological development that may have lasting consequences for cognitive and socioemotional development.
