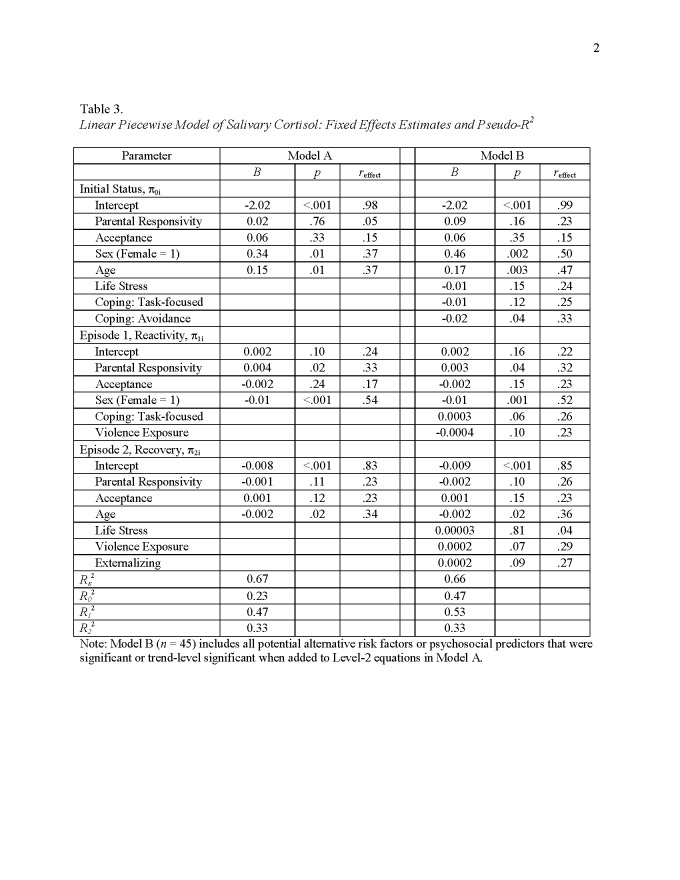# Correction: Selective Impact of Early Parental Responsivity on Adolescent Stress Reactivity

**DOI:** 10.1371/annotation/9294793a-c267-46db-94de-16a3b6705a1e

**Published:** 2013-04-10

**Authors:** Daniel A. Hackman, Laura M. Betancourt, Nancy L. Brodsky, Lara Kobrin, Hallam Hurt, Martha J. Farah

There were errors in Tables 1 and 3. The correct versions of the tables can be found here:

Table 1: 

**Figure:**
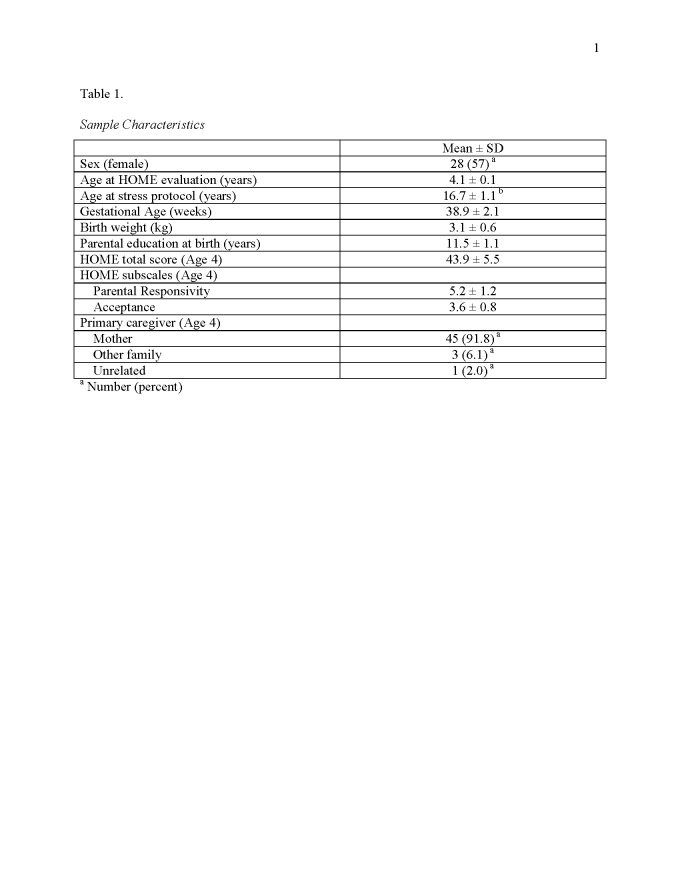


Table 3: 

**Figure:**